# Prevalence and determinants of depression up to 5 years after colorectal cancer surgery: results from the ColoREctal Wellbeing (CREW) study

**DOI:** 10.1111/codi.15949

**Published:** 2021-11-25

**Authors:** Lynn Calman, Joshua Turner, Deborah Fenlon, Natalia V. Permyakova, Sally Wheelwright, Mubarak Patel, Amy Din, Jane Winter, Alison Richardson, Peter W. F. Smith, Claire Foster, members of the CREW Study Advisory Committee, Jo Armes, Janis Baird, Andrew Bateman, Nick Beck, Graham Moon, Claire Hulme, Peter Hall, Karen Poole, Susan Restorick‐Banks, Paul Roderick, Claire Taylor, Jocelyn Walters, Fran Williams, Lynn Batehup, Jessica Corner, Deborah Fenlon

**Affiliations:** ^1^ School of Health Sciences University of Southampton Southampton UK; ^2^ College of Human and Health Sciences Swansea University Swansea UK; ^3^ Faculty of Medicine University of Southampton Southampton UK; ^4^ Division of Health Sciences University of Warwick Warwick UK; ^5^ University Hospitals Southampton NHS Trust Southampton UK; ^6^ Social Statistics and Demography Social Sciences University of Southampton Southampton UK

**Keywords:** colorectal cancer, determinants, depression, quality of life, risk factors

## Abstract

**Aim:**

Depression experienced by people with colorectal cancer (CRC) is an important clinical problem affecting quality of life. Recognition of depression at key points in the pathway enables timely referral to support. This study aimed to examine depression before and 5 years after surgery to examine its prevalence and identify determinants.

**Method:**

The ColoREctal Wellbeing (CREW) study is a prospective UK cohort study involving 872 adults with nonmetastatic CRC recruited before surgery with curative intent. Questionnaires completed before surgery and 3, 9, 15, 24, 36, 48 and 60 months after surgery captured socio‐demographics and assessed depression (Centre for Epidemiologic Studies Depression Scale, CES‐D) and other psychosocial factors. Clinical details were also gathered. We present the prevalence of clinically significant depression (CES‐D ≥ 20) over time and its predictors assessed before and 2 years after surgery.

**Results:**

Before surgery, 21.0% of the cohort reported CES‐D ≥ 20 reducing to 14.7% 5 years after surgery. Presurgery risk factors predicting subsequent depression were clinically significant depression and anxiety, previous mental health service use, low self‐efficacy, poor health, having neoadjuvant treatment and low social support. Postsurgery risk factors at 2 years predicting subsequent depression were clinically significant depression, negative affect, cognitive dysfunction, accommodation type and poor health.

**Conclusion:**

Depression is highly pervasive in people with CRC, exceeding prevalence in the general population across follow‐up. Our findings emphasize the need to screen and treat depression across the pathway. Our novel data highlight key risk factors of later depression at important and opportune time points: before surgery and at the end of routine surveillance. Early recognition and timely referral to appropriate support is vital to improve long‐term psychological outcomes.

**What does this paper add to the literature?**

Depression in people living with colorectal cancer is an important clinical problem. Our study shows that the prevalence of depression exceeds prevalence in the general population over time. It also highlights risk factors for later depression at important time points (before surgery and at the end of routine follow‐up) which informs strategies for recognition and referral to appropriate psychosocial support.

## INTRODUCTION

Depressive disorders are one of the highest contributors to the global burden of disease, affecting 4% of people [1]. The prevalence of depression is greater in people living with cancer (rates range between 8% and 27% [2]) and thus it is a clinical and policy priority [3]. In people living with colorectal cancer (CRC), the prevalence of depression is also greater when compared with the general population, with rates reaching 37% [4, 5, 6, 7]. Depression in people living with cancer is associated with poor quality of life (QoL) [8], reduced adherence to treatment [9] and reduced survival [10] and is associated with an increased risk of suicide [11]. More specifically, people with CRC and depression have poorer QoL, health status and wellbeing after diagnosis and surgery [5, 7].

Despite this, depression is often not identified in people with cancer, and few are treated [6], possibly due to a range of factors associated with underreporting of mental health issues (e.g. stigma) [12]. Timely recognition and referral to support and intervention is recommended in international clinical guidelines for depression [13], with UK guidelines recommending targeting screening in people at most risk [14]. However, determinants of depression in people living with cancer are poorly characterized [15] with calls for more evidence in people with CRC [16]. Identification of people most at risk also informs the development of interventions, reduces disease burden and improves planning of psychosocial care resources [17].

Several determinants of depression in people with CRC have been described, including female gender [4, 18, 19], low socio‐economic status [20], higher stage disease [21], receipt of neoadjuvant and adjuvant treatments [22] and low social support [23, 24]. Findings related to age are inconsistent [19, 21, 25] but may be due to sample characteristics (e.g. recruitment of an older sample [25]). Depressive symptoms in people with CRC are also reported to reduce over time [4, 5] but much research is cross‐sectional [16]. We conducted a scoping review and found that no published longitudinal studies in people with nonmetastatic CRC have examined presurgery risk factors associated with reporting depression up to 5 years after treatment (Appendix 1).

UK clinical guidance recommends before and after treatment as key time points to appraise psychological outcomes in people living with cancer [26]. Assessment close to diagnosis allows for a better understanding of the impact of cancer and its treatment on depression outcomes [15, 26]. Early screening is also encouraged due to its benefits: improving treatment adherence, reducing the burden on health services and patients, enhancing communication between clinical teams and patients and more timely referral to support [17, 27]. Addressing psychological outcomes after treatment gives patients the opportunity to reflect on the impact and psychosocial concerns following the intense scheduling of cancer treatment [15, 26]. Posttreatment CRC surveillance is valuable to provide reassurance, as patients feel greater concern when its frequency decreases [28], possibly due to reduced contact with clinical teams [29], so we highlight this time point as being important. In the UK it is recommended that routine surveillance appointments cease after 2 years [30] with some variation in international guidelines [31].

This paper presents analysis from the ColoREctal Wellbeing (CREW) study [32], a UK prospective cohort study investigating factors associated with the recovery of health and wellbeing following CRC. Data were collected before and at regular intervals up to 5 years after surgery. Data comprised socio‐demographic, clinical information and patient‐reported outcomes examining a selection of psychosocial variables (including depression) informed by a conceptual framework of recovery following cancer diagnosis and treatment [33]. The analysis assesses ‘clinically significant levels’ of depression via self‐reporting, and whilst this is not a ‘clinical diagnosis’ of depression, which requires a comprehensive assessment accounting for contextual factors [14], the cut‐off used has high concordance with psychiatric interviews [34] suggesting the experience of high levels of depressive symptomology [35]. In this paper we describe the prevalence of clinically significant levels of depression from before surgery up to 5 years after surgery and, given the levels of depression before and at 2 years after surgery, identify which characteristics are associated with subsequent clinically significant levels of depression up to 5 years after surgery.

## METHOD

### Study sample

The CREW study is a prospective cohort study of adults (≥18 years) with nonmetastatic CRC (Dukes A–C) treated by surgery with curative intent. Inclusion and exclusion criteria are published elsewhere [32].

### Data collection

Details of study procedures have been previously reported [32]. Eligible participants were recruited from 29 UK National Health Service (NHS) centres between November 2010 and March 2012. Participants consented and completed questionnaires before surgery (baseline). Follow‐up questionnaires were posted at regular intervals (3, 9, 15, 24 months and then annually up to 5 years after surgery). Clinical and treatment information was gathered from NHS medical databases at participating centres. Ethical approval was granted by the UK NHS NRES Committee South Central – Oxford B (REC ref. 10/H0605/31). Information collected in the study did not inform the care of the participants involved due to the study design and anonymization of the data.

### Measures

Patient‐reported depression was captured using the 20‐item Centre for Epidemiologic Studies Depression Scale (CES‐D) [36]. Higher scores indicate greater levels of depression (range 0–60). A recent meta‐review demonstrated that CES‐D is responsive to change and suitable for screening for depression in people with cancer [37].

A score of ≥20 has previously been used in studies involving people with cancer to define a ‘clinically significant level’ of depression [38] and has been shown to be highly concordant with psychiatric interviews [34]. A recent meta‐analysis examining the screening accuracy of CES‐D noted the ≥20 cut‐off to be more appropriate than the standard ≥16 cut‐off [35]. Thus the ≥20 cut‐off was selected as an indicator of a clinically significant levels of depression for this study, but this does not constitute a formal diagnosis of clinical depression.

### Determinants/covariates

Table 1 lists the validated patient‐reported outcome measures, socio‐demographic questions and clinical information captured before surgery and 2 years after surgery which were used as covariates in the analyses. Covariates are presented according to the conceptual framework domains [33] and the rationale for each measure is provided elsewhere [32]. Validated measures were repeated at every time point unless otherwise indicated. Selection of covariates, including European Organisation for Research and Treatment of Cancer (EORTC) subscales, was informed by our scoping review (Appendix 1). Alongside depression, accommodation type, health status (EQ‐5D), age and ethnicity were found to be significantly associated with participant attrition in the CREW study [39] and were included in the model to account for this.

**TABLE 1:** CREW study measures presented by conceptual framework domains [33] for regression analysis

Domain	Characteristic of interest	Measure
Preexisting factors (socio‐demographics)	Age	
	Gender	
	Ethnicity	
	Employment status^a^	
	Accommodation type^a^	
	Deprivation index	Index of Multiple Deprivation (IMD) [61]
Clinical factors	Tumour site	
	Dukes stage^a^	
	Neoadjuvant treatment^a^	
	Surgery type	
	Adjuvant treatment^a^	
	Stoma status	
	Number of comorbidities^b^	Self‐reported measure [40]
	Previous use of mental health services^c^	
Environmental factors	Domestic status	
	Life events	List of Threatening Experience Questionnaire (LTE‐Q) [62]
	Social support	Medical Outcomes Study ‐ Social Support Survey (MOS‐SSS) [63]
Personal factors	Self‐efficacy	Self‐efficacy for Managing Chronic Disease (SEMCD) scale [64]
		Cancer Survivors' Self‐Efficacy Scale (CS‐SES) [65]
	Affect	Positive and Negative Affect Schedule Short Form (PANAS‐SF) [66]
Psychosocial outcomes	State anxiety^d^	State‐Trait Anxiety Inventory, State scale (STAI‐S) [67]
	Wellbeing	Personal Wellbeing Index–Adult (PWI‐A) [68]
	Health status	EuroQoL, five dimensions three levels (EQ−5D−3L) [69]
	Quality of life (QoL)^e^	Quality of Life in Adult Cancer Survivors (QLACS) scale [70]: Cancer‐Specific Summary Score (QLACS‐CSS), Benefit of Cancer (QLACS‐BC)
	Symptoms and functioning^b^	European Organization for Research and Treatment of Cancer quality of life measure (EORTC QLQ‐C30) [71]: function scales: physical, emotional, cognitive, social; symptom scales: fatigue, pain, insomnia, financial worry

^a^ To avoid imprecise estimates from the low counts in the regression analyses two or more groups were merged together: unemployed and retired (employment status); renting and other (accommodation type); Stages C1 and C2 (Dukes stage); radiotherapy, chemotherapy and both (neoadjuvant treatment; adjuvant treatment).

^b^ Collected from 3 months onwards. Selection of EORTC subscales was informed by previous work involving people with CRC [7, 22, 24, 25].

^c^ Self‐reported at baseline only.

^d^ We used a cut‐off of ≥40 to indicate a clinically significant level of anxiety [72].

^e^ Items comprising the QLACS‐CSS and QLACS‐BC were collected from 9 months onwards.

### Statistical analysis

The total CES‐D score was summarized at each time point using its median and interquartile range (IQR) to examine changes over time. The number and proportion of participants reporting clinically significant levels of depression (CES‐D ≥ 20) were also assessed over time.

Two multivariable logistic regression models were fitted to predict clinically significant levels of depression up to 5 years after surgery (Appendix 2): model 1 included depression together with other covariates collected before surgery (baseline); model 2 included depression together with other covariates collected at 2 years after surgery. Multicollinearity was assessed in each model using the variance inflation factor (VIF). The VIF ranged from 1.05 to 2.18 for model 1 and from 1.08 to 2.51 for model 2. A VIF below 10 indicates that there was no evidence of multicollinearity in our models.

Missing data were imputed according to published guidelines for the measures selected. If unavailable, these were omitted from the final model. The number of comorbidities was first assessed at 3 months but was included in model 1 due to its stability over time [40].

A population‐average approach was applied to account for the time‐varying nature of the binary outcome, where each model was adjusted for the clustering of observations within the participants [41]. Regression analyses were based on a backwards elimination of statistically nonsignificant predictors. The significance level was fixed at 5% and all analyses were completed in Stata 14.

## RESULTS

### Sample characteristics

**FIGURE 1:**
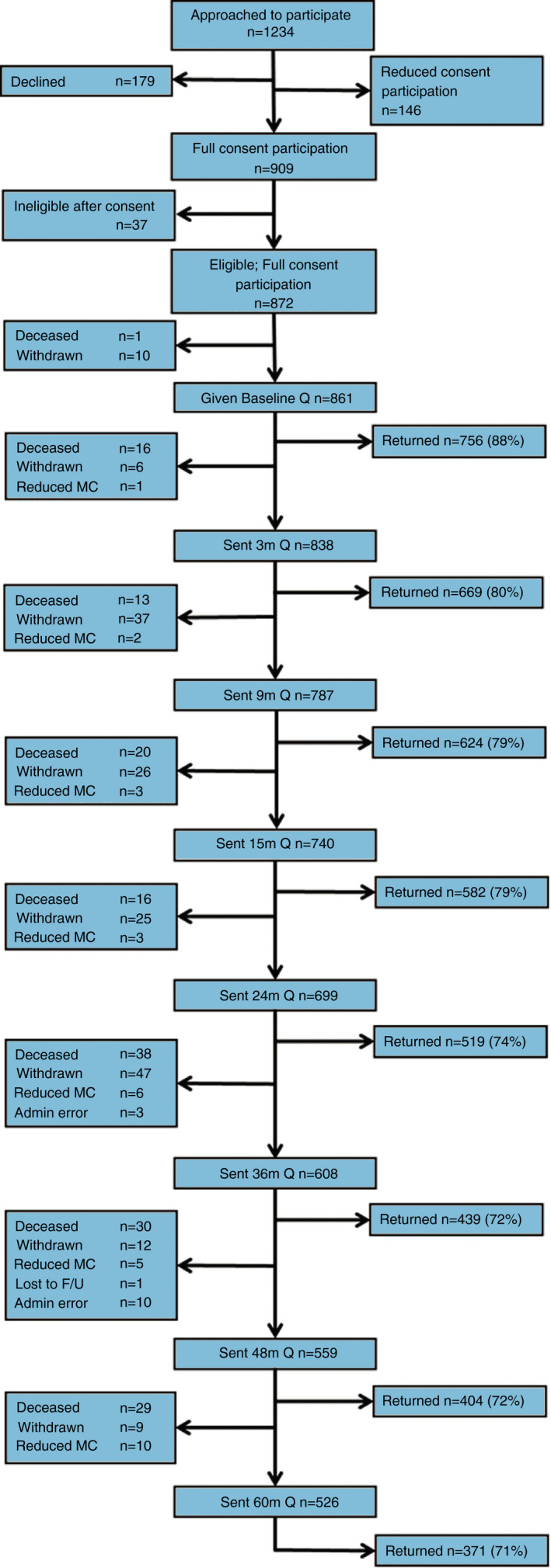
CREW study participant flowchart. Note: participants who were not sent a questionnaire because of mental capacity issues or through administrative error remained eligible for the questionnaire at the next time point. Definitions: full consent, participants consented to questionnaire follow‐up and the collection of medical details; reduced consent, participants consented to the collection of medical details only. Abbreviations: F/U, follow‐up; MC, mental capacity; Q, questionnaire

One thousand and eighteen participants were recruited into CREW and 872 consented to questionnaire follow‐up. Figure 1 presents the participant flow over follow‐up; full details of study recruitment and descriptive statistics are published elsewhere [39, 42]. The sample was representative of the eligible patients treated during the recruitment period [39, 42]. Table 2 shows the demographic and clinical characteristics of the 741 participants who returned a baseline questionnaire and had completed the CES‐D; these participants had a mean age of 67.54 years (SD = 10.26 years). Over 54% of the sample underwent laparoscopic surgery and 40% underwent open surgery for CRC.

**TABLE 2:** Sociodemographic and clinical information comparisons of CES‐D < 20 and ≥20 reported at baseline (*N* = 741)

Covariates reported at baseline	*n* (%)	CES‐D < 20, *n* (%)	CES‐D ≥ 20, *n* (%)	*p*‐value (chi‐square test)
Age groups (years)				
50 or younger	47 (6.4%)	32 (68.1%)	15 (31.9%)	0.073
51–60	113 (15.3%)	83 (73.5%)	30 (26.5%)	
61–70	285 (38.6%)	236 (82.8%)	49 (17.2%)	
71–80	217 (29.4%)	173 (79.7%)	44 (20.3%)	
81 or older	77 (10.4%)	58 (75.3%)	19 (24.7%)	
Gender				
Male	440 (59.4%)	373 (84.8%)	67 (15.2%)	**<0.001**
Female	301 (40.6%)	210 (69.8%)	91 (30.2%)	
Ethnicity				
White British	623 (92.7%)	491 (78.8%)	132 (21.2%)	0.898
Other ethnic group	49 (7.3%)	39 (79.6%)	10 (20.4%)	
Deprivation (IMD) quintile				
1st quintile (least deprived)	146 (20.1%)	123 (84.2%)	23 (15.8%)	0.086
2nd quintile	150 (20.6%)	123 (82%)	27 (18%)	
3rd quintile	142 (19.5%)	113 (79.6%)	29 (20.4%)	
4th quintile	136 (18.7%)	99 (72.8%)	37 (27.2%)	
5th quintile (most deprived)	153 (21%)	114 (74.5%)	39 (25.5%)	
Domestic status				
Married/living with partner	524 (71.1%)	430 (82.1%)	94 (17.9%)	**<0.001**
Single/widowed/divorced/separated	213 (28.9%)	150 (70.4%)	63 (29.6%)	
Employment status				
Employed	201 (27.3%)	158 (78.6%)	43 (21.4%)	0.980
Unemployed/retired	535 (72.7%)	421 (78.7%)	114 (21.3%)	
Accommodation type				
Owner occupied	589 (79.9%)	473 (80.3%)	116 (19.7%)	**0.021**
Renting/other^a^	148 (20.1%)	106 (71.6%)	42 (28.4%)	
Previous use of mental health services				
No	670 (94.5%)	536 (80%)	134 (20%)	**<0.001**
Yes	39 (5.5%)	22 (56.4%)	17 (43.6%)	
Tumour site				
Colon	475 (64.4%)	374 (78.7%)	101 (21.3%)	0.911
Rectum	263 (35.6%)	208 (79.1%)	55 (20.9%)	
Dukes stage				
A	109 (14.7%)	93 (85.3%)	16 (14.7%)	0.335
B	391 (52.8%)	303 (77.5%)	88 (22.5%)	
C (C1 and C2)	229 (30.9%)	178 (77.7%)	51 (22.3%)	
Could not be determined^b^	11 (1.5%)	9 (81.8%)	2 (18.2%)	
Neoadjuvant treatment (any type)				
No	592 (80.7%)	465 (78.5%)	127 (21.5%)	0.649
Yes	142 (19.3%)	114 (80.3%)	28 (19.7%)	
Surgery type^c^				
Laparoscopic	401 (54.3%)	–	–	–
Open	299 (40.5%)	–	–	
Not available	38 (5.2%)	–	–	
Adjuvant treatment (any type)^c^				
No	477 (64.6%)	–	–	–
Yes	261 (35.4%)	–	–	
Stoma^c^				
No	262 (35.9%)	–	–	–
Yes	468 (64.1%)	–	–	
Number of comorbidities^d^				
0	168 (27.6%)	143 (85.1%)	25 (14.9%)	.055
1	194 (31.9%)	160 (82.5%)	34 (17.5%)	
2	144 (23.6%)	107 (74.3%)	37 (25.7%)	
3+	103 (16.9%)	78 (75.7%)	25 (24.3%)	

Note

*p*‐values in bold indicate a statistically significant difference at the 5% level.

*Abbreviation:* IMD, Index of Multiple Deprivation.

^a^ Other accommodation includes: temporary accommodation, living in residential or nursing home, living with others (e.g. friends or family).

^b^ Dukes stage could not be determined for 11 full consent patients with small tumours following neoadjuvant therapy.

^c^ Captured from the medical records after baseline.

^d^ Self‐reported at 3 months.

### Depression over time

At baseline (before surgery), people who were women, single, living in rented accommodation and had previously used mental health services were more likely to report clinically significant CES‐D scores (Table 2).

Median scores peaked before surgery at 12.0 (IQR = 11.7) and decreased to 9.5 (IQR = 12.0) at 5 years (Table 3). Similarly, the proportion of participants reporting clinically significant levels of depression also peaked before surgery at 21.0% and reduced to 14.7% at 5 years (Table 3). Overall, 303 participants (34.8%) reported clinically significant depression at least once during their 5 years of follow‐up.

**TABLE 3:** Descriptive statistics for the CES‐D score and clinically significant level of depression (CES‐D ≥ 20) at each time point from before surgery to 5 years after surgery

	Presurgery	Postsurgery						
	Baseline	3 months	9 months	15 months	24 months	36 months	48 months	60 months
*N*	741	642	605	534	483	382	369	319
CES‐D total, median (IQR)	12.0 (11.7)	11.1 (12.0)	10.0 (13.0)	9.0 (12.0)	9.0 (10.9)	8.0 (11.7)	9.0 (11.0)	9.5 (12.0)
CES‐D ≥ 20, *n* (%)	158 (21.3)	124 (19.3)	106 (17.5)	70 (13.1)	73 (15.1)	49 (12.8)	48 (13.0)	47 (14.7)

*Abbreviations:* CES‐D, Centre for Epidemiologic Studies Depression Scale; IQR, interquartile range

### Presurgery determinants of clinically significant levels of depression

Table 4 presents only the significant presurgery factors associated with the likelihood of reporting a clinically significant level of depression.

**TABLE 4:** Multivariable logistic regression model of clinically significant depression (CES‐D ≥ 20) up to 5 years after surgery, significant covariates collected before surgery (baseline)

Theme block	Covariates	OR	95% CI	*p*‐value
Socio‐demographic factors	Age groups (years)			
	50 or younger	Ref.	–	–
	51–60	0.50	0.22–1.10	0.086
	61–70	0.50	0.26–0.97	**0.040**
	71–80	0.55	0.27–1.13	0.103
	81 or older	0.77	0.33–1.80	0.550
Clinical and treatment factors	Tumour site			
	Colon	Ref.	–	–
	Rectum	0.55	0.35–0.87	**0.011**
	Neoadjuvant treatment			
	None	Ref.	–	–
	Yes, any therapy	2.99	1.75–5.09	**<0.001**
	Previous use of mental health services			
	No	Ref.	–	–
	Yes	3.33	1.81–6.12	**<0.001**
	Unknown	0.65	0.23–1.81	0.411
Environmental factors	Domestic status			
	Married/living with a partner	Ref.	–	–
	Single/widowed/divorced/separated	2.02	1.32–3.09	**0.001**
Personal factors	Self‐efficacy (SEMCD)			
	Low confidence	Ref.	–	–
	Moderate confidence	0.42	0.24–0.73	**0.002**
	Confident	0.35	0.20–0.61	**<0.001**
	Very confident	0.18	0.08–0.37	**<0.001**
Psychosocial factors	Depression (CES‐D)			
	<20	Ref.	–	–
	≥20 (clinical level)	3.44	2.18–5.45	**<0.000**
	Anxiety (STAI‐S)			
	<40	Ref.	–	–
	≥40 (high level)	1.82	1.15–2.87	**0.010**
	Social support (MOS‐SSS)			
	<100 (not full)	Ref.	–	–
	=100 (full)	0.41	0.23–0.74	**0.003**
	Health status (EQ‐5D‐3L)			
	Not perfect health	Ref.	–	–
	Perfect health	0.42	0.24–0.75	**0.003**

Note

*p*‐values in bold indicate a statistically significant difference at the 5% level. The model controls for the time point of the outcome report (postsurgery 3–60 m), which was statistically significant.

*Abbreviations:* CES‐D, Centre for Epidemiologic Studies Depression Scale; EQ‐5D‐3L, EuroQoL five dimensions, three levels; MOS‐SSS, Medical Outcome Study Social Support Scale; SEMCD, Self‐Efficacy for Managing Chronic Disorders Scale; STAI‐S, State‐Trait Anxiety Inventory, State scale.

Participants who reported clinically significant levels of depression before surgery had a higher risk of being depressed over follow‐up (OR = 3.44, 95% CI = 2.18–5.45); this was similar for highly anxious people (OR = 1.82, 95% CI = 1.15–2.87). People with a low level of self‐efficacy (confidence) to manage the consequences of a chronic condition were also at a greater risk of reporting clinically significant levels of depression. Conversely, people who reported ‘full’ social support (OR = 0.41, 95% CI = 0.23–0.74) had lower odds of reporting clinically significant depression, and this was also the case for ‘perfect’ health status (OR = 0.42, 95% CI = 0.24–0.75).

A greater risk of reporting clinically significant levels of depression up to 5 years after surgery was found in people who underwent neoadjuvant treatment (OR = 2.99, 95% CI = 1.75–5.09) and in those who reported previous use of mental health services (OR = 3.33, 95% CI = 1.48–5.24) compared with those who did not. People with rectal cancer also had lower odds of having clinically significant depression compared with those with colon cancer (OR = 0.55, 95% CI = 0.35–0.87).

Both age and domestic status were found to be statistically significant predictors of subsequent clinically significant depression. Younger participants (<51 years old) were at greater risk of experiencing clinically significant levels of depression when compared with people aged 61–70 years (OR = 0.50, 95% CI = 0.26–0.97), although this was not evident when compared with other age groups (Table 4). The odds of reporting clinically significant levels of depression were twice as high for people who did not have a partner (OR = 2.02, 95% CI = 1.32–3.09) compared with those who did.

### Determinants 2‐years after surgery

Table 5 presents only the significant predictors, captured 2 years postsurgery, of clinically significant levels of depression reported at 2 years. Similar to the presurgery situation, participants reporting clinically significant levels of depression at 2 years were at greater risk of subsequent depression up to 5 years (OR = 3.14, 95% CI = 1.41–7.04). Those who had higher scores for negative affect were also at greater risk (OR = 1.21, 95% CI = 1.08–1.36).

**TABLE 5:** Multivariable logistic regression model of clinically significant depression (CES‐D ≥ 20) up to 5 years after surgery, significant covariates collected at 2 years

Theme block	Covariates	OR	95% CI	*p*‐value
Socio‐demographic factors	Accommodation type			
	Owner occupied	Ref.	–	–
	Rented/other	2.38	1.23–4.62	**0.010**
Personal factors	Affect (PANAS‐SF)			
	Negative affect	1.21	1.08–1.36	**0.001**
Psychosocial factors	Depression (CES‐D)			
	<20	Ref.	–	–
	≥20 (clinical level)	3.14	1.41–7.04	**0.005**
	Health status (EQ‐5D‐3L)			
	Not perfect health	Ref.	–	–
	Perfect health	0.28	0.12–0.68	**0.005**
	Wellbeing (PWI‐A)			
	≥70 (good)	Ref.	–	–
	<70 (poorer)	2.40	1.25–4.61	**0.008**
	Cognitive functioning (EORTC QLQ‐C30)			
	No problem	Ref.	–	–
	Some problem	2.21	1.03–4.77	**0.043**

Note

*p*‐values in bold indicate a statistically significant difference at the 5% level.

*Abbreviations:* CES‐D, Centre for Epidemiologic Studies Depression Scale; EORTC‐QLQ‐C30, European Organisation for Research and Treatment of Cancer Quality of Life Core‐30 Questionnaire; EQ‐5D‐3L, EuroQoL five dimensions, three levels; PANAS‐SF, Positive and Negative Effect Schedule Short Form; PWI‐A, Personal Wellbeing Index – Adult.

People reporting problems with cognitive function (OR = 2.21, 95% CI = 1.03–4.77) and poorer wellbeing (OR = 2.40, 95% CI = 1.25–4.61) at 2 years also had higher odds of experiencing clinically significant depression later. Participants who did not own their accommodation were also at greater risk of reporting clinically significant depression (OR = 2.38, 95% CI = 1.23–4.62).

In contrast, the risk of reporting clinically significant levels of depression was lower amongst those who had ‘perfect’ health status at 2 years (OR = 0.28, 95% CI = 0.12–0.68).

## DISCUSSION

This is the first prospective cohort study to examine the prevalence and risk factors associated with clinically significant levels of depression in people with nonmetastatic CRC assessed before and up to 5 years after surgery. Our results reveal that clinically significant levels of depression remain a long‐term problem for a considerable proportion of people, despite median CES‐D scores reducing over time from initial diagnosis. These results are consistent with previous findings [4, 5]. For example, our prevalence rates across each time point occur within the range observed by cross‐sectional studies of people living with CRC (7%−37% [4, 5, 6, 7]) and are considerably higher than the median prevalence found in the general population (CES‐D ≥ 20; 11.8%) [35].

The novelty of this study is the investigation of risk factors of clinically significant levels of depression at two key time points in the cancer care pathway as recommended by UK clinical guidance [26]: close to diagnosis (before surgery) and when posttreatment routine surveillance ends (2 years after surgery). Identifying risk factors improves planning of psychosocial care and informs the development of interventions [17]. We identified several pre‐ and postsurgery risk factors of depression consistent with previous work [4, 5, 7, 19, 20, 22, 23, 24, 25].

Importantly, our findings underscore the need for depression screening close to diagnosis, with clinically significant levels before surgery being identified as a risk for later depression. Early screening has been shown to have a positive impact on care by leading to more timely referrals for psychological intervention [17, 27]. Our analysis at 2 years after surgery also suggests the need for assessment of depression and depressive symptomology (negative affect) when posttreatment surveillance ends. Regular appraisal of psychological needs throughout the pathway aligns with recent emphasis on risk stratification in the UK NHS Long Term Plan to inform personalized care for people with cancer and facilitate referral to appropriate levels of care [43]. Psychosocial interventions for people with CRC have been reported to be beneficial in improving symptomology of depression and anxiety, as well as QoL [44, 45]. Novel strategies for follow‐up have been tested in Australia [46] and Canada [47] and are being considered in the USA [48]. Such strategies can help target specialist resources as these become increasingly scarce [48]. Innovative models of psychological screening and care (e.g. stepped care and nurse‐led collaborative interventions) for people with cancer are effective in reducing psychological symptoms, improve QoL for people with a depressive or anxiety disorder and are cost‐effective [49, 50]. Internationally, variability in models and approaches to survivorship care and complexity in reimbursement for psychosocial and integrated care make implementation a challenge [51].

Our presurgery analysis also highlights at‐risk groups to whom we should direct depression screening. People undergoing neoadjuvant treatment commonly face more complex surgery, stoma formation, additional side effects and increased treatment time [22], which can explain our findings and so attention should focus on this group. People with rectal cancer had a lower risk of clinically significant depression over time but no previous CRC studies have reported tumour site as a significant predictor of depression [16]. This relationship was also unexpected, as people with rectal cancer often have complex treatment regimens (including neoadjuvant treatment) [52] which may impact psychological outcomes, particularly those who later have a permanent stoma [53]. One possible explanation could be that a more complex treatment pathway may result in greater contact with clinical teams and this may improve perceptions of support [29, 54] that could help to reduce depression symptomology. Nevertheless, this finding requires further investigation.

Our analysis further recommends that depression screening should target people with a history of mental health problems or with psychological comorbidities (e.g. anxiety). This is unsurprising, as the level of anxiety tends to peak close to diagnosis [55] and it commonly co‐occurs with depression [8].

The value of assessing self‐efficacy and social support early in the pathway was highlighted by our presurgery analysis. This is important given the increasing role of self‐management for people with cancer [56]; thus confidence to manage consequences of cancer and its treatment need to be assessed early on. Assessing the level of social support at the point of diagnosis is imperative given its importance for depression outcomes and later QoL [23].

Our analysis at 2 years after surgery highlighted other at‐risk groups in whom assessment and support for depression may be helpful. People with cognitive difficulties postsurgery were at greater risk of later depression; this is important as cognitive dysfunction is a commonly reported consequence of CRC treatment [57]. However, caution should be applied as it can be difficult to delineate cognitive dysfunction as a result of cancer treatment or as a symptom of depression and/or anxiety [58]. Type of accommodation (rented or other) was also highlighted as a risk factor, but this specifically has not been reported previously. It could be used as a descriptor of socio‐economic status which has been noted to be a risk factor for anxiety, depression and distress in people with cancer [20, 59]. This highlights the need for additional support for this group as low socio‐economic status may indicate a low availability of resources important for coping, which may result in poorer psychological outcomes [59].

Study strengths include the scale and representative nature of the CREW sample with over 91% of all eligible patients approached to participate [39, 42]. Loss to follow‐up is expected in cohort studies but our response rates remained high up to 5 years (Figure 1; 71%). Participants who withdrew by 5 years were more likely to report clinically significant depression, were ≥80 years of age, did not own accommodation (renting or other) and were of non‐White ethnicity at baseline [39]. Therefore, our findings may underestimate the true prevalence of depression among CRC cancer survivors in the UK. Additionally, our sample represents patients from one type of healthcare system (the UK NHS) whereby access to and provision of specialist services is universal and free at the point of delivery.

Patient‐reported depression may not account for contextual factors considered in diagnostic interviews [14]. Nevertheless, the cut‐off used suggested clinically significant levels of depressive symptomology [35] and is highly concordant with psychiatric interviews [34]. A high prevalence of depression over time may be attributed to its undertreatment [6]. However, due to a high level of missing data, as a result of poor self‐reporting of health service use in CREW, we were unable to examine use of psychological treatment, which may explain our findings. We examined our mental health service use data in a bivariate analysis with CES‐D scores for interest (Appendix 3).

Scoping of the literature (Appendix 2) identifies this is as one of the first studies to include a presurgery assessment on a range of socio‐demographic, psychosocial and clinical factors and the only one to collect data for up to 5 years afterwards to examine risk factors of clinically significant levels of depression. The importance of this work is highlighted by the dearth of evidence examining the long‐term psychological impacts in people living with and beyond cancer [3], including people with CRC [16]. The need for research into the short‐ and long‐term psychological impacts of cancer and its treatment has been identified as a Top 10 research priority in the UK [60] and our analysis contributes knowledge to this for two crucial time points in the CRC care pathway.

In summary, our results indicate that depression is an enduring problem in people with nonmetastatic CRC even at 5 years after surgery. Before surgery it affects one in five people and one in seven people at 5 years after surgery, both of which are higher than reported in the general population. Our findings clearly highlight the need for screening for depression across the pathway to improve depression outcomes in the long term. Early screening should be focused on those with a history of mental health issues, high levels of anxiety, low self‐efficacy, poor health status and low levels of support, whilst clinicians should also monitor people who undergo neoadjuvant treatment. The end of routine oncology surveillance is also an opportune time to assess symptoms of depression, especially as the frequency of contact with clinical teams decreases. At this time point, assessment should focus on people with poor health, a lower socio‐economic status and problems from treatment (e.g. cognitive dysfunction). Depression in people living with cancer is associated with poor health and wellbeing and has an impact on survival and adherence to treatment, so early recognition and treatment may lead to overall improved outcomes for patients.

## CONFLICT OF INTERESTS

LC has received an honorarium for teaching from Boehringer Ingelheim. DF has received an honorarium for teaching from Roche.

## AUTHOR CONTRIBUTIONS

CF, DF, PWS conceived and obtained funding for the study. LC, JW, AR and the Study Advisory Committee made substantial contributions to the development and design of this work. NP and MP analysed the data with the support of PWS. JT, LC, DF, NP, PWS, SW, JW and CF contributed to interpretation of data. JT drafted the manuscript with substantial contributions from LC, CF and SW. All authors provided critical comments on drafts of the manuscript and approved the final manuscript.

## ETHICAL APPROVAL

The study was approved by the UK National Health Service National Research Ethics Service (REC reference number: 10/H0605/31). Informed consent was obtained from all individual participants involved in the study.

## Data Availability

The data underlying this article will be shared on reasonable request to the corresponding author. Information regarding data access is available at http://horizons‐hub.org.uk/access_data.html

## APPENDIX 1 Scoping review search strategy and key studies identified which involve people with CRC including at least one follow‐up time point

Dates literature searches conducted:

- October 2016
- August 2017
- September 2018
- May 2019
- July 2019
- October 2020
- March 2021

Databases searched:

- CINHAL
- APA PsycINFO
- APA PsycARTICLES
- MEDLINE (EBSCO)
- ISI Web of Science

Limiters:

- Date published: 1 January 2010 to 1 October 2020
- English language
- Human studies
- Peer‐reviewed

Search terms:

Subject/MeSH headings used where appropriate

**Table:**

Colorectal neoplasms OR Colorectal cancer
(Colon OR Rectum) AND (neoplasms OR cancer)
Depression OR MM Depression
Anxiety OR MH Anxiety Disorder
‘Mental Health’
‘Psychological disorder’
‘Psychological distress’
‘Worry’
MH Stress, Psychological
MH Mental Disorders
MH Fear

**Table:**

Lead author and year	Country of study	Sample	Assessment time points	Depression measure	Key findings	Comparison with the CREW study
Dunn et al., 2013 [1]	Australia	1884 CRC survivors; Stages I–IV	T1: 5 months after diagnosis. Follow‐up: 12 (T2), 24 (T3), 36 (T4), 48 (T5) and 60 (T6) months postdiagnosis	BSI‐18	• Four trajectories of depressive symptoms: constant low levels, constant high levels and people who increase from low and those who reduce from high levels • Men, younger participants, later stage, poor social support and lower education were more likely to experience high levels of depression • 16.1% of participants were in the ‘constant high’ level trajectory for depressive symptoms (BSI‐18 Depression Subscale)	• No presurgery assessment of psychological distress •Recruitment of patients with metastatic CRC
Hart and Charles, 2013 [2]	USA	139 CRC patients (Stages I–IV)	T1: presurgery. Follow‐up: T2, 6 months; T3, 12 months; T4, 18 months postsurgery	CES‐D	• Mean (SD): T1, 10.45 (8.11); T2, 9.33 (7.80); T3, 9.41 (8.74); T4, 9.49 (9.28) • Older adults reported lower levels of depressive symptoms. Men had fewer depressive symptoms than women	• The prevalence of clinical levels of depression was not assessed • No follow‐up assessment beyond 18 months postsurgery • Recruitment of patients with metastatic CRC
Gonzalez‐Saenz de Tejada et al., 2017 [3]; Quintana et al., 2018 [4]	Spain	972 CRC patients (including patients in relapse)	T1: presurgery. Follow‐up: T2, 12 months; T3, 24 months postsurgery	HADS	• 19.6% of participants reported depression at T1 • Patients with depression improved less than participants not reporting depression or anxiety in all health‐related quality of life domains (EORTC QLQ‐C30) • Overall, few differences in depression symptoms in people undergoing either open or laparoscopic surgery • Mean (SD) (laparoscopy vs. open): T1, 4.28 (4.12) vs. 5.33 (4.84); T2, 3.52 (3.85) vs. 4.08 (4.31); T3, 3.50 (3.97) vs. 4.28 (4.38)	• Recruitment of patients with metastatic CRC • Recruitment of patients in relapse (CREW excluded patients with previous cancer diagnosis) • No follow‐up assessment beyond 24 months postsurgery
Mols et al., 2018 [5]	Netherlands	315 CRC survivors (Stages I–IV)	Annual follow‐up (1–4 years): T1, 2010; T2, 2011; T3, 2012; T4, 2013	HADS	• Significantly higher prevalence of depression (19.0%, *N* = 2625) compared with a matched population (12.8%, *N* = 315) during their first assessment • Reduction in depression symptoms over time with the largest difference identified when examining the first and fourth assessments (mean change −0.89) • Fewer depressive symptoms were reported in people who were older, low QoL and lower physical, role, cognitive, emotional and social functioning	• Participants recruited 1 to 4 years postdiagnosis • Recruitment of participants with metastatic CRC

*Abbreviations:* BSI–18, Brief Symptom Inventory−18; CES‐D, Centre for Epidemiologic Studies Depression Scale; CRC, colorectal cancer; EORTC QLQ‐C30, European Organisation for Research and Treatment of Cancer Quality of Life Core‐30 Questionnaire; HADS, Hospital Anxiety and Depression Scale; QoL, quality of life.

## APPENDIX 2 Availability of the covariates at two time points of the regression analyses

**Table:**

Thematic block	Topic/measure	Taken time points in separate regression models	
		Baseline (presurgery)	2 years postsurgery
Preexisting factors (socio‐demographics)	Age	+	+
	Gender	+^a^	+^a^
	Ethnicity	+^a^	+^a^
	Employment status	+	+
	Accommodation type	+	+
	Index of multiple deprivation	+^1^	+^1^
Clinical factors	Tumour site	+	+
	Dukes stage	+	+
	Neoadjuvant treatment	+	+
	Adjuvant treatment	−	+
	Surgery type	−	+
	Stoma status	−	+
	Number of comorbidities	+^n^	+
	Previous use of mental health services	+	‐
Environmental factors	Domestic status	+	+
	Life events	−	+
	Medical Outcome Study Social Support Scale (MOS‐SSS)	+	+
Personal factors	Self‐Efficacy for Managing Chronic Disease 6‐Item Scale (SEMCD)	+	−
	Cancer Survivor Self‐Efficacy Scale (CS‐SES)	−	+
	Positive and Negative Affect Schedule Short Form (PANAS‐SF)	+	+
Psychosocial outcomes	Centre for Epidemiologic Studies Depression Scale (CES‐D)	+	+
	Quality of Life in Adult Cancer Survivors (QLACS) scale: *QLACS Cancer‐Specific Summary Score*	−	+
	*QLACS Benefit of Cancer subscale*	−	+
	State‐Trait Anxiety Inventory, State scale (STAI‐S)	+	+
	Personal Wellbeing Index – Adult (PWI‐A)	+	+
	EQ‐5D‐3L	+	+
	EORTC‐QLQ‐C30: physical functioning	−	+
	EORTC‐QLQ‐C30: emotional functioning	−	+
	EORTC‐QLQ‐C30: cognitive functioning	−	+
	EORTC‐QLQ‐C30: social functioning	−	+
	EORTC‐QLQ‐C30: fatigue	−	+
	EORTC‐QLQ‐C30: pain	−	+
	EORTC‐QLQ‐C30: insomnia	−	+
	EORTC‐QLQ‐C30: financial worry	−	+

*Note:* data are taken from the same time point unless otherwise annotated: ^a^data taken from baseline time point, ^b^data taken from the 3 month follow‐up time point.

*Abbreviations:* EORTC QLQ‐C30, European Organisation for Research and Treatment of Cancer Quality of Life Core‐30 Questionnaire; EQ‐5D‐3L, EuroQoL five dimensions, three levels.

Key: ‘+’ included in regression analysis for the time point; ‘−’ indicates excluded from regression analysis for the time point due to the measure not being assessed at the time point.

## APPENDIX 3 Self‐reported health service use (Have you used any of the following health and social services in the last 12 months?)

**Table:**

Time point (postsurgery)	24 months		36 months		48 months		60 months	
CES‐D score	≥20, *n* (%)	<20, *n* (%)	≥20, *n* (%)	<20, *n* (%)	≥20, *n* (%)	<20, *n* (%)	≥20, *n* (%)	<20, *n* (%)
*N*	73	410	49	333	48	321	47	272
Mental health services	2 (2.7)	0 (0)	2 (4.1)	1 (0.3)	3 (6.3)	1 (0.3)	1 (2.1)	1 (0.4)
Counselling services	4 (5.5)	2 (0.5)	6 (12.2)	2 (0.6)	2 (4.2)	5 (1.6)	1 (2.1)	2 (0.7)
Psychiatrist	4 (5.5)	2 (0.5)	4 (8.2)	1 (0.3)	3 (6.3)	0 (0)	2 (4.3)	1 (0.4)
Self‐help group	5 (6.8)	4 (1.0)	2 (4.1)	5 (1.5)	3 (6.3)	6 (1.9)	3 (6.4)	4 (1.5)

*Abbreviation:* CES‐D, Centre for Epidemiologic Studies Depression Scale.
